# Dyke–Davidoff–Masson Syndrome in Pediatrics: Case Report of Atypical Status Epilepticus and Therapeutic Challenges in the Caribbean

**DOI:** 10.1155/carm/8388244

**Published:** 2025-09-24

**Authors:** María Belén Martín-Sanz, Delvis Lucas-Muñoz, Manuel Colomé-Hidalgo, Gabriela Vásquez-Gómez

**Affiliations:** ^1^Department of Medical Specialties and Public Health, Research Group of Humanities and Qualitative Research in Health Science, Rey Juan Carlos University, Alcorcón, Spain; ^2^Department of Pediatric Neurology, Dr. Hugo Mendoza Pediatric Hospital, Santo Domingo, Dominican Republic; ^3^Department of Public Health, Autonomous University of Santo Domingo (UASD), Santo Domingo, Dominican Republic

**Keywords:** case report, cerebral hemiatrophy, Dyke–Davidoff–Masson syndrome (DDMS), status epilepticus

## Abstract

Dyke–Davidoff–Masson syndrome (DDMS), also known as cerebral hemiatrophy, is characterized by brain damage resulting in hypoplasia of one cerebral hemisphere. It is described as a rare disorder, primarily characterized by epileptic seizures and convulsions, as well as hemiparesis and cognitive impairments. We present the evolution of a child with DDMS and the neurodevelopmental milestones achieved from the onset of symptoms at 10 days of age to 3 years. Clinical features included persistent seizures and hemiparesis. Neuroimaging revealed right cerebral hemiatrophy with associated structural changes. The aim of this study is to present a case report of a pediatric patient with DDMS, detailing the clinical evaluation, therapeutic approaches, and disease progression, while describing the healthcare process and challenges associated with managing this rare condition in the Dominican Republic. Through this, we aim to improve the therapeutic strategies implemented in the region for the management of this infrequent condition and enhance the understanding of DDMS in the Caribbean.

## 1. Introduction

The Dyke–Davidoff–Masson Syndrome (DDMS), also known as cerebral hemiatrophy, is a rare neurological condition that was first described in 1933 and results from a brain injury during fetal development or early childhood [1, 2]. This condition leads to the loss of neurons and hypoplasia in one of the cerebral hemispheres, adversely affecting proper brain growth. Clinical manifestations include seizures, hemiparesis, facial asymmetry, intellectual disability, language disorders, and learning difficulties. Typical radiological findings include unilateral cerebral atrophy, which varies according to the severity of the injury and the age of the patient [1–5]. The syndrome is classified as congenital (associated with vascular anomalies in the internal carotid artery and middle cerebral artery) and acquired (due to risk factors such as atherosclerosis or carotid stenosis) [1]. Regarding the pathophysiology of DDMS, hypotheses such as cerebral hypoperfusion and venous congestion have been proposed.

In this report, we present the case of a three-year-old male patient diagnosed with DDMS, who experienced seizures 10 days after birth. Our study aims to illustrate the evolution of the case in order to increase the limited documentation regarding this rare syndrome by informing on the neurodevelopmental milestones achieved by the patient. The authors have not identified any previously reported cases with this symptomatology in our region. Through this, we aim to improve the therapeutic strategies implemented in the Dominican Republic for the management of this infrequent condition.

## 2. Patient Information and Clinical Findings

Three-year-old male patient. The patient was referred to neurology at 6 months of age due to generalized clonic seizures. The patient is seen in the neurology clinic at 8 months of age after being referred for generalized clonic involuntary movements lasting less than 1 minute during sleep. He was born at 37 weeks of gestation. At approximately 6 weeks of gestation, the mother experienced pelvic pain suggestive of a threatened abortion. The episode was managed with progesterone therapy, rest, and outpatient follow-up, with no additional complications reported throughout the remainder of the pregnancy. The mother did not undergo any prenatal screening, as there was no awareness of any risk factors or warning signs during the pregnancy that would have prompted such evaluation. The pregnancy was considered low-risk at the time, and no clinical indication for further testing was identified. He was delivered by cesarean section due to a double nuchal cord and weighed 3.2 kg, with regular intake of hematinic supplements, and was discharged stable alongside his mother at birth. Regarding his family history, he has a paternal cousin with Patau's syndrome. At 10 days of age, he experienced three convulsive episodes within a 24 h period, lasting between 30 s and 1 min. He was evaluated by his physician, monitored, and discharged due to the absence of clinical findings. He subsequently remained seizure-free until 6 months of age. At 6 months of age, he began experiencing epileptic seizures during sleep that persisted throughout the night. These seizures were characterized as focal tonic seizures with impaired awareness, typically involving supraversion of gaze and, occasionally, vocalizations. They occurred daily, showed a clear nocturnal predominance, and were not associated with sphincter relaxation. They occurred daily, showed a clear nocturnal predominance, and were frequently triggered by afternoon naps. He also presented with microcephaly, psychomotor delay, and left convergent strabismus with epicanthus, for which he was referred to pediatric neurology for further evaluation. Between six and 8 months of age, he did not receive medical care or treatment due to prolonged waiting times for a public pediatric neurology appointment and inability to access private consultation. During this period, seizures persisted and increased in both frequency and intensity, with two episodes lasting longer than 5 minutes, until his first pediatric neurology visit at 8 months of age. At the 8-month evaluation by pediatric neurology, the physical examination revealed global hyperreflexia, hypertonia, and an inability to maintain head support. The patient had a short neck, a flattened nasal bridge, low-set ears, and a broad nasal base. There was muscular weakness in both upper and lower extremities, particularly significant in the left hemibody. The thorax appeared symmetrical, with rhythmic heart sounds and a soft, nondistended abdomen. He exhibited a micropenis and redundant foreskin. The patient was awake and alert but showed limited connection with the examiner, responding to voice and exhibiting symmetrical facial expressions. His fists were clenched. An ophthalmological evaluation revealed symmetrical bilateral optic nerves with normal vascularization, normal retinal attachment, normal macular appearance, reactive pupils, and a clear anterior chamber. Auditory evoked potential tests showed no abnormalities. An abdominal ultrasound showed no significant alterations. In the context of recurrent anemia and the absence of prior newborn screening, an evaluation of glucose-6-phosphate dehydrogenase (G6PD) enzyme activity was performed. Another factor that prompted this assessment was the patient's predisposition to frequent gastrointestinal infections, which could potentially require treatment with oxidizing medications. Additionally, given the patient's African descent—a population with a higher prevalence of this enzymatic deficiency—G6PD testing was clinically indicated. The enzymatic activity was measured using a quantitative assay, which confirmed G6PD deficiency. Genetic testing was subsequently conducted, revealing hemizygosity for a pathogenic variant in the G6PD gene. These findings were reported alongside an activated partial thromboplastin time (APTT) of 46 s.

### 2.1. Neurodevelopmental Milestones

**Figure 1:**
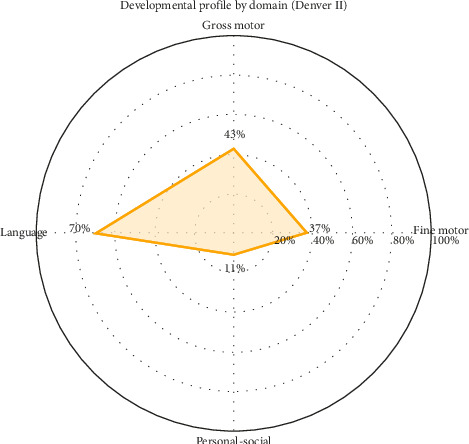
Developmental profile by domain.

A developmental assessment was conducted using the Denver II Developmental Screening Test, which compares the expected age of milestone acquisition with the actual attainment age across four key domains: personal-social, fine motor-adaptive, language, and gross motor skills. Significant delays were observed in milestones such as bringing hands to the midline (achieved at 12 months; expected at 3 months), throwing a ball (24 months; expected at 18 months), running well (32 months; expected at 24 months), identifying body parts (20 months; expected at 14 months), and combining two words (28 months; expected at 24 months). Additionally, several milestones, including climbing stairs without assistance, pedaling a tricycle, and buttoning clothing, had not yet been achieved.  Table 1 presents a comparison between the expected age of acquisition and the patient's actual attainment age for each milestone according to the Denver II scale.  Figure 1 presents a radar chart illustrating the percentages of developmental milestones achieved in each domain relative to the 100% expected for chronological age. The final score was calculated by assigning one point for normal milestones, 0.5 points for delayed milestones, and 0 points for unachieved milestones. Greater impairment is observed in the personal-social domain (11%), followed by fine motor-adaptive (37%) and gross motor (43%), while language demonstrates the highest relative performance (70%). The overall profile is consistent with global developmental delay, with predominant involvement of the personal-social domain.

## 3. Diagnostic Assessment

**Figure 2:**
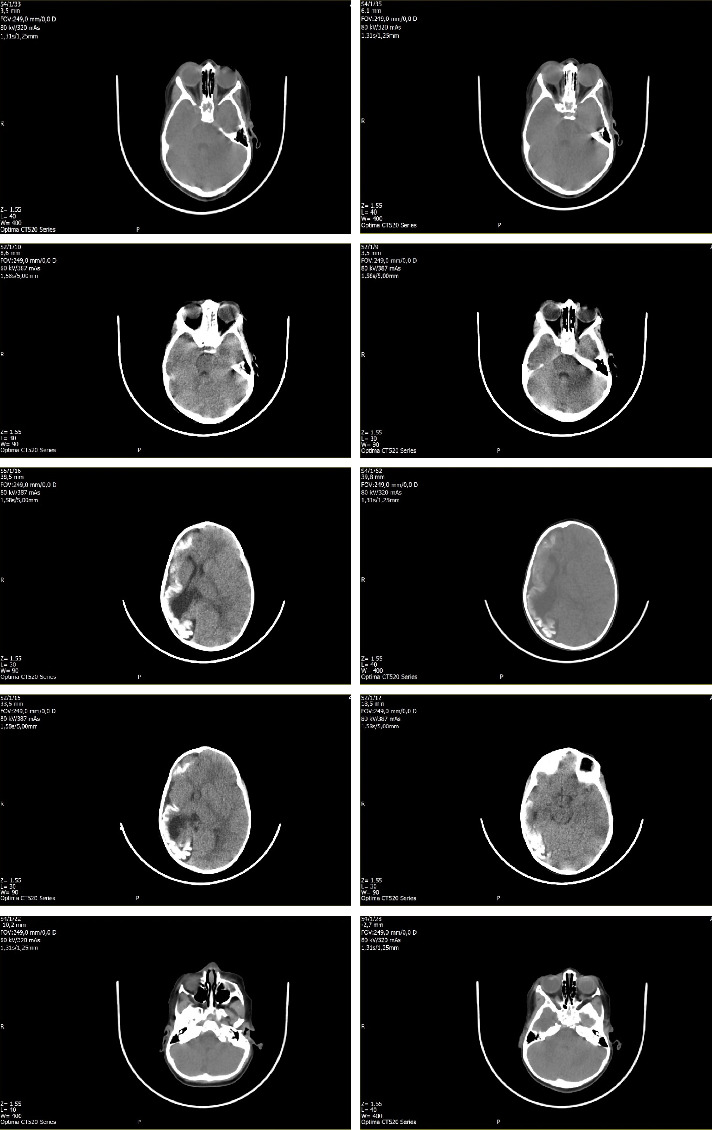
Findings of cranial tomography.

Up to 12 months of age, the initial diagnosis was psychomotor delay, as imaging studies and differential diagnosis could not be performed due to access barriers, including limited infrastructure, insufficient professionals, and bureaucratic challenges in test authorization. At 1 year of age, a cranial tomography with 3 mm axial cuts was performed, revealing findings of volume loss in the right cerebral hemisphere, as shown in Figure 2, along with calcifications in the white matter. There was dilation of the ipsilateral lateral ventricle and a shift of the midline to the right. The left cerebral hemisphere appeared normal in size. The loss of right-hemispheric parenchyma disrupts the corticospinal tracts, resulting in contralateral hemiparesis and delays in motor and adaptive milestones; ex vacuo ventricular dilatation and parenchymal calcifications confirm an early, chronic brain injury consistent with the patient's progressive psychomotor delay. Preservation of the left hemisphere largely spares language networks, producing the asymmetric clinical pattern characteristic of DDMS. The imaging diagnosis was discussed at a multidisciplinary conference with paediatric neuroradiologists and the hospital's neuropaediatrics service, and both teams agreed on the neuroradiological findings and their clinical correlation. The final confirmed diagnosis was structural epilepsy associated with DDMS.

**Figure 3:**
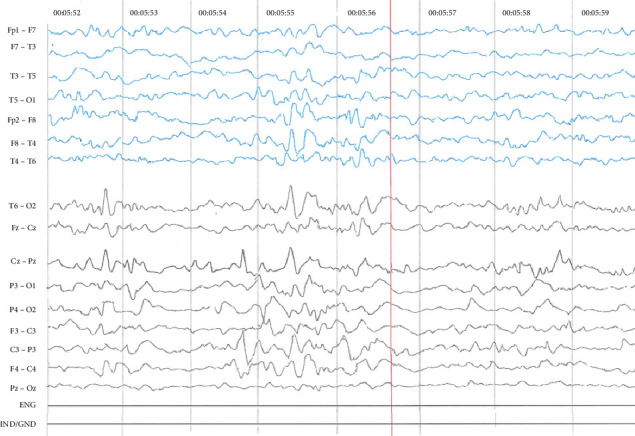
Findings of digital electroencephalogram.

At 2 years of age, a digital electroencephalogram was conducted using the international 10/20 system (Figure 3). This was the patient's first EEG, which had been initially indicated after the first clinical evaluation but was delayed because the parents could not afford the cost at that time. The study consisted of a one-hour digital EEG including both wakefulness and spontaneous sleep. The results showed disorganized, slow, and diffuse baseline activity of a mild to moderate degree, with a predominant frequency in the theta-delta range. During drowsiness, the dominant rhythm was posterior at 3 to 4 Hz. During sleep, there was an absence of frequency and amplitude gradient with abundant epileptiform activity during the tracing. Pathological graphoelements were observed, characterized by focal onset sharp waves and slow waves in the right temporoparietal region, along with rapid interhemispheric synchronization.

## 4. Therapeutic Interventions

The patient has been receiving pharmacological treatment and physical therapy since the time of diagnosis.

Physical therapy is conducted weekly using neurorehabilitation techniques based on the Bobath and Vojta methods.

Pharmacological management was guided by seizure semiology, patient age and sex, and the selection of agents effective against both focal and generalized seizures. Preference was given to drugs with favorable pharmacokinetic profiles, low interaction potential, and ease of administration to optimize adherence and minimize adverse effects. Treatment has consisted of the continuous daily administration of antiseizure medication since diagnosis. Initial therapy with magnesium valproate was associated with increased seizure frequency. Subsequent trials with carbamazepine, lamotrigine, and topiramate proved ineffective. Given the persistence of nocturnal seizures, levetiracetam and oxcarbazepine, both at appropriate doses, were introduced sequentially, resulting in an approximate 60% reduction in seizure frequency. Finally, clonazepam was added, achieving partial but clinically meaningful seizure control. Additionally, risperidone was prescribed to manage behavioral symptoms, and baclofen was added to address contralateral hemibody spasticity. Semiannual evaluations are conducted to adjust medication dosages in accordance with changes in body weight. The diagnosis of DDMS is characterized by the refractoriness of epileptic seizures to antiseizure medications; therefore, polytherapy remains necessary as part of the management approach to optimize seizure control in this condition. Given the complexity and refractoriness of this case, close clinical follow-up and multidisciplinary monitoring are essential to ensure effective long-term management and timely therapeutic adjustments.

## 5. Clinical Progress and Timeline

At present, at 3 years of age, the patient continues to experience persistent seizures, now occurring on a weekly basis and of shorter duration compared to the initial presentation. Sleep has been identified as the primary trigger, with episodes most frequently occurring during the afternoon nap. Persistent irritability and emotional dysregulation are also noted, and he is awaiting evaluation by early intervention services. He attends preschool but demonstrates difficulties in peer relationships, as well as challenges with concentration and focused attention. Treatment adherence is maintained, as reported by caregivers, without unexpected side effects.

**Figure 4:**
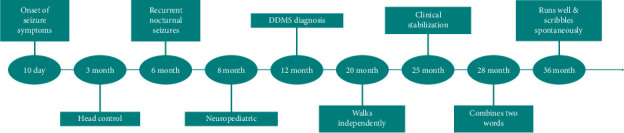
Key clinical and neurodevelopmental milestones.

From a motor perspective, he walks independently with a hemiparetic gait but is not yet able to ascend or descend stairs without assistance. Linguistically, he can repeat simple sentences and name familiar objects, interacting primarily within the family environment. Key clinical and neurodevelopmental milestones are summarized in Figure 4.

## 6. Discussion

DDMS is a rare condition with an unknown incidence [1]. It was first described in 1933 [2], and fewer than 250 cases have been reported worldwide since then [3, 4]. This pathology is more frequent in males and predominantly affects the left hemisphere [5]. The clinical manifestations of this syndrome are varied, with a history of seizures/epilepsy present in 76% of cases, which are more common in the early years of life, as seen in our patient [6]. Gökce et al. [7] describe the classic triad of DDMS: epilepsy, hemiplegia/hemiparesis, and motor and cognitive delay. The course of the disease varies depending on the clinical manifestations and the type of DDMS, which can be congenital or acquired [1, 8]. Our study outlines the progression and developmental milestones of a patient with congenital DDMS. In such cases, seizures are mostly tonic-clonic and generalized, as in our patient, compared to focal seizures in the acquired type [9]. Prognosis is less favorable when symptom onset occurs before 2 years of age and when patients experience recurrent or prolonged seizures despite treatment [5, 9–11]. Previous studies suggest the hypothesis that increased blood flow in the contralateral hemisphere during early years may exacerbate cerebral neurodegeneration [1]. Additionally, emotional dysregulation and irritability, as reported in this case, have also been described in patients with DDMS [12–14].

As described in previous studies, the diagnosis, as in this case, is based on clinical examination and neuroimaging findings [1, 15, 16]. These images typically reveal cerebral hemiatrophy, which is present in 100% of cases, along with parenchymal changes and dilation of the ipsilateral lateral ventricle, accompanied by compensatory changes in the ipsilateral skull, such as hemicranial thickening (71.4% of cases) or hyperpneumatization of the paranasal sinuses (71.4% of cases) [1, 6, 17]. Differential diagnoses include Sturge–Weber syndrome, linear nevus sebaceous syndrome (LNSS), Silver–Russell syndrome, Fishman syndrome, and Rasmussen encephalitis [17, 18].

Currently, the pharmacological management of DDMS lacks standardized protocols and, in clinical practice, is generally based on symptomatic regimens with antiepileptic drugs [1, 4]. While in our case initial therapy with magnesium valproate was ineffective, cases of DDMS controlled with monotherapy, such as those described by Al-Smair et al. (valproic acid) [3], Khan et al. (levetiracetam) [4], and Behera et al. (oral carbamazepine) [19], have been reported, highlighting that therapeutic response in DDMS can vary substantially between patients.

Subsequently, in our patient, the combination of levetiracetam and oxcarbazepine, supplemented with clonazepam, led to a partial reduction in seizure frequency, in line with the findings of Rondão et al., who emphasized that responses are often partial despite polytherapy [9]. Similarly, Alam et al. [5] reported two adolescents with DDMS treated chronically with carbamazepine and levetiracetam, with the addition of valproic acid in one case, also achieving partial clinical improvement. Zawar et al. [20] described an infant with DDMS who was treated with intravenous phenobarbital, phenytoin, and midazolam, followed by oral valproic acid, initially achieving seizure control but later experiencing recurrence of focal seizures. Complementarily, Li et al. [21] described an adolescent-onset DDMS case treated with magnesium valproate and carbamazepine, achieving a significant reduction in seizures limited to brief partial episodes during 24 months of follow-up. This finding supports the role of such pharmacological combinations in the partial control of seizures in DDMS, although variability in clinical response depends on context and individual characteristics.

Finally, it is important to note that in our context, limiting factors such as the lack of liquid carbamazepine formulations and the economic constraints characteristic of the Caribbean region significantly influenced therapeutic decisions and underscore the need to adapt pharmacological regimens to real-world drug availability and local healthcare resources. Overall, our case illustrates the complexity of DDMS management, where individualized polytherapy is essential and seizure control is often partial rather than complete, consistent with what has been described in the specialized literature [3–5, 9, 21].

In addition to pharmacological treatment, following prior recommendations, a multidisciplinary approach is necessary, incorporating rehabilitation therapies such as physical therapy, occupational therapy, and psychological care [8, 22]. Multidisciplinary management is particularly complex in the Dominican Republic due to the lack of awareness of this condition in healthcare settings and the challenges in diagnosis and holistic care. Currently, no legislation supports rare diseases in the Dominican Republic, although there is a High-Cost Medication Program (PMAC) that allows patients with these conditions to access medication [23]. Families caring for children with rare diseases in this context face numerous challenges, including social and economic difficulties, as well as the impact and high cost of managing the disease [24–26].

This case highlights several key learning points related to DDMS in pediatric patients. First, it underscores the importance of recognizing the characteristic clinical presentation and neuroimaging findings, as early identification is essential for accurate diagnosis and adequate management. Additionally, it illustrates the therapeutic challenges posed by the frequent refractoriness of epileptic seizures associated with this syndrome, even with the appropriate administration of antiseizure medications, reinforcing the need for close and continuous follow-up.

In the specific context of the Caribbean, this case also draws attention to the limited access to specialized diagnostic studies and certain advanced pharmacological options, which necessitates prioritizing locally available treatments and fostering multidisciplinary approaches to optimize care in resource-constrained settings. It further highlights the importance of adapting international diagnostic and therapeutic protocols to regional realities in order to maintain a patient-centered approach.

In conclusion, we emphasize the importance of continuing to expand the knowledge base and reporting of these low-prevalence cases to enhance the understanding of healthcare management and outcomes in documented cases. This highlights significant gaps in the understanding of this pathology and the challenges associated with its treatment in the Caribbean. Addressing these gaps will contribute to advancing academic and clinical knowledge and improving the quality of care for patients with DDMS.

## Figures and Tables

**Table 1:** Comparison of expected versus patient achievement age for each milestone using the Denver II.

Domain	Milestone	Expected age (months)	Patient age (months)	Interpretation
Gross motor	Head control	2	9	Delayed
	Hands together in midline	3	12	Delayed
	Sits without support	6	11	Delayed l
	Walks independently	12	20	Delayed
	Throws ball	18	24	Delayed
	Runs well	24	36	Delayed
	Climbs stairs without help	24	—	Not achieved
	Pedals tricycle	36	—	Not achieved

Fine motor-adaptive	Grasps rattle	3	4	Delayed
	Transfers object hand-to-hand	6	7	Delayed
	Scribbles spontaneously	14	36	Delayed
	Builds a bridge with cubes	36	—	Not achieved

Language	Babbles	6	4	Normal
	“Mama”/“Dada” specific	10	10	Normal
	One additional word	12	12	Normal
	Points to familiar objects	12	12	Normal
	Knows body parts	14	20	Delayed
	Combines two words	24	28	Delayed
	Uses three-word sentences	36	—	Not achieved
	States first and last name	35	35	Normal
	Identifies 3 colors	36	—	Not achieved
	Speech intelligible to strangers	36	36	Normal

Personal-social	Social smile	2	2	Normal
	Eats cracker independently	6	—	Not achieved
	Plays peek-a-boo	7	—	Not achieved
	Drinks from cup unassisted	12	—	Not achieved
	Indicates needs without crying	11	—	Not achieved
	Uses spoon independently	16	—	Not achieved
	Removes clothes	20	—	Not achieved
	Puts on shoes (no laces)	24	—	Not achieved
	Buttons clothing	30	—	Not achieved
	Washes hands independently	36	—	Not achieved
